# *In Situ* Shell‐Isolated Nanoparticle‐Enhanced Raman Spectroscopy of Nickel‐Catalyzed Hydrogenation Reactions

**DOI:** 10.1002/cphc.201901162

**Published:** 2020-02-04

**Authors:** Caterina S. Wondergem, Josepha J. G. Kromwijk, Mark Slagter, Wilbert L. Vrijburg, Emiel J. M. Hensen, Matteo Monai, Charlotte Vogt, Bert M. Weckhuysen

**Affiliations:** ^1^ Inorganic Chemistry and Catalysis Group Debye Institute for Nanomaterials Science Utrecht University Universiteitsweg 99 3584 CG Utrecht The Netherlands; ^2^ Laboratory of Inorganic Materials and Catalysis Eindhoven University of Technology P.O. Box 513 5600 MB Eindhoven The Netherlands

**Keywords:** heterogeneous catalysis, in situ spectroscopy, nickel, Raman spectroscopy, SHINERS

## Abstract

Synthesis methods to prepare lower transition metal catalysts and specifically Ni for Shell‐Isolated Nanoparticle‐Enhanced Raman Spectroscopy (SHINERS) are explored. Impregnation, colloidal deposition, and spark ablation have been investigated as suitable synthesis routes to prepare SHINERS‐active Ni/Au@SiO_2_ catalyst/Shell‐Isolated Nanoparticles (SHINs). Ni precursors are confirmed to be notoriously difficult to reduce and the temperatures required are generally harsh enough to destroy SHINs, rendering SHINERS experiments on Ni infeasible using this approach. For colloidally synthesized Ni nanoparticles deposited on Au@SiO_2_ SHINs, stabilizing ligands first need to be removed before application is possible in catalysis. The required procedure results in transformation of the metallic Ni core to a fully oxidized metal nanoparticle, again too challenging to reduce at temperatures still compatible with SHINs. Finally, by use of spark ablation we were able to prepare metallic Ni catalysts directly on Au@SiO_2_ SHINs deposited on a Si wafer. These Ni/Au@SiO_2_ catalyst/SHINs were subsequently successfully probed with several molecules (*i. e*. CO and acetylene) of interest for heterogeneous catalysis, and we show that they could be used to study the *in situ* hydrogenation of acetylene. We observe the interaction of acetylene with the Ni surface. This study further illustrates the true potential of SHINERS by opening the door to studying industrially relevant reactions under *in situ* or *operando* reaction conditions.

## Graphical abstract

**Sparking the reaction**: Spark ablation is presented as a new method for the preparation of working lower transition metal catalysts supported on Shell‐Isolated Nanoparticle‐Enhanced Raman Spectroscopy (SHINERS)‐active Au@SiO_2_ nanoparticles. The gas phase hydrogenation of acetylene is studied with *in situ* SHINERS over the prepared Ni catalysts, reporting the first non‐noble metal catalysis study with SHINERS.

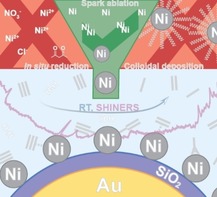

## 1 Introduction

Heterogeneous catalysis is one of the most industrially interesting areas in chemistry as solid catalysts are involved in over 80 % of all manufacturing processes in the chemical industry.^1^ Therefore, understanding the workings of solid catalysts at the nanoscale has been and remains a hot topic of research.^2^ Small improvements in existing catalysts’ efficiency, selectivity, or activity can have a tremendous effect on industrial processes, whereas unravelling active sites may lead to the development of altogether new heterogeneous catalysts. In order to do this, structure‐performance relationships need to be established through which we can determine the role of *e. g*. different facets on a catalyst nanoparticle (NP) in adsorption, conversion, and desorption of the reagent of interest. As such chemical processes take place on the catalyst surface, many studies involving surface science methods have been dedicated to the interaction of molecules with metal surfaces.^3^, ^4^ These studies were traditionally carried out on surfaces of well‐defined single crystal planes at low temperatures and pressures, but often these results cannot be directly translated to industrially employed heterogeneous catalysts.^5^ First of all, (metal) NPs consist of an array of different facets, and second, the different temperatures and pressures employed have been observed to induce dynamic changes in the facets present on nanoparticles, and influence interaction of the reagent of interest itself.^6^, ^7^, ^8^ Therefore, characterization studies of working catalysts are required to gain more insight into actual structure‐performance relationships involving the so‐called *operando* spectroscopy approach.^9^, ^10^

Vibrational spectroscopy is extremely suitable to achieve this goal, since it provides molecular fingerprints of both surface and gas‐phase species present during catalytic reactions and can be employed under a wide range of experimental conditions (*i. e*. elevated temperatures and pressures). For example, *operando* infrared (IR) spectroscopy has been used to investigate the ammoxidation of propane over a rutile‐SbVO_4_ catalyst,^11^ alcohol oxidation over Au^12^ and Cu‐TEMPO^13^ catalysts, the water‐gas‐shift reaction over Pt/Al_2_O_3_ 14 and NO_x_ reduction over a Pt−Rh/Ba/Al_2_O_3_ catalyst,^15^ while a recent example from our group focused on the understanding of structure‐sensitivity of CO_2_ hydrogenation over supported Ni catalysts.^16^ Complementary to IR spectroscopy, Raman spectroscopy offers the additional possibility to directly monitor metal‐adsorbate interactions, whereas these vibrations are generally not IR‐active.^17^ Furthermore, *operando* Raman spectroscopy has been widely used to investigate metal (oxide) catalysts over a wide variety of chemical reactions, including the investigation of Pt(O_x_) catalysts supported on SiO_2_, Al_2_O_3_ and CeO_2_ under reducing and oxidizing conditions,^18^ the selective oxidation of propylene to acrolein over V_2_O_5_/Nb_2_O_5_ catalysts,^19^ and the influence of doping a NiO catalyst with Fe in the hydrogen and oxygen evolution reactions.^20^

Although traditional Raman spectroscopy suffers from low detection limits, the use of the signal enhancing technique Surface‐Enhanced Raman Spectroscopy (SERS) has been reported to allow detection of single molecules.^21^, ^22^, ^23^, ^24^ By employing Au or Ag NPs that exhibit Localized Surface Plasmon Resonance (LSPR) upon illumination with (laser) light of the right frequency, strong electromagnetic fields can be induced near the surface of the NPs. This makes SERS an incredibly suitable tool for surface studies, since it only enhances signals of species on or close to the catalyst surface.

To enable application of SERS in *in situ* and *operando* heterogeneous catalysis, the Au and Ag NPs need to be stabilized and physically separated from the catalyst under investigation by coating the Au and Ag NPs with a thin layer of dielectric oxide, like SiO_2_. This technique, Shell‐Isolated Nanoparticle‐Enhanced Raman Spectroscopy (SHINERS)^25^ is rapidly developing into a very useful and practical method for *in situ* surface studies of catalytic reactions.^26^, ^27^ The biggest limitation for such Shell‐Isolated Nanoparticles (SHINs) to be generally applied in the field of catalysis is their moderate thermal stability (∼450 °C). Studies are thus so far limited to noble metal catalysts, which are easily reduced to their metallic, active form, without the need for high temperatures.^28^ Some recent examples from our own group involve the hydrogenation of CO into higher hydrocarbons, including alcohols, over SHINERS‐active Rh/Au@SiO_2_ and RhFe/Au@SiO_2_ catalysts,^29^ as well as the hydrogenation of phenylacetylene into styrene and ethylbenzene over SHINERS‐active Pt/Au@SiO_2_.^30^

However, noble metals, such as Pt, Pd and Rh, are not always preferred in chemical industry due to their high cost and low natural abundancy. Instead, metals that are more abundant and cost‐efficient are often employed, but these often need more rigorous temperature treatment to reach their active catalytic phase. For example, although Pt is a more active hydrogenation catalyst and is used for *in situ* SHINERS hydrogenation studies, Ni is often used in industrial hydrogenation due to the much lower cost (1560 €/kg vs. 10 €/kg, respectively; Pd: 2700 €/kg^31^). Reduction of Ni precursors requires high temperatures, which can have a detrimental effect on the enhancing properties of Au@SiO_2_ SHINs, in turn making SHINERS studies on such materials extremely challenging.

In this paper, we will explore various methods of catalyst/SHIN preparation based on Ni as a showcase of lower transition metals, to enable *in situ* SHINERS investigation of industrially relevant chemical reactions. We will demonstrate the challenges in the preparation of active Ni/Au@SiO_2_ catalyst/SHIN systems in both previously reported preparation methods (*i. e*., the Precursor (Pr) and Colloidal (Col) synthesis methods). Next, we will present a new synthesis method for the preparation of active Ni catalysts on Au@SiO_2_ SHINs by Spark Ablation (SA).^32^ We will then test the different Ni‐based catalysts with various probe molecules, such as CO, phenylacetylene, and acetylene, to assess their suitability for SHINERS studies. Finally, we will demonstrate that we can observe hydrogenation of acetylene with *in situ* SHINERS over Ni(SA)/Au@SiO_2_.

## 2 Results and Discussion

### 2.1 Preparation and Characterization of Ni/Au@SiO_2_ Catalyst/Shell‐Isolated Nanoparticles

**Figure 1:**
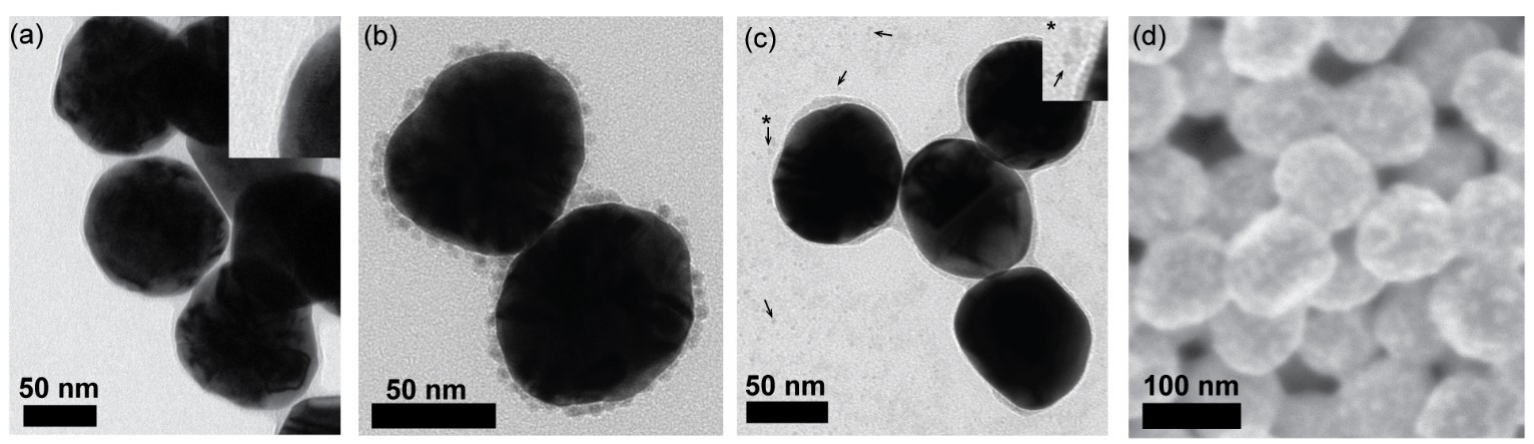
Characterization data for Au@SiO_2_ Shell‐Isolated Nanoparticles (SHINs) prepared via the Precursor (Pr), Colloidal Deposition (CoI) and Spark Ablation (SA) method. (a) Transmission Electron Microscopy (TEM) image of Au@SiO_2_. A 90 nm Au core surrounded by a 2 nm SiO_2_ shell can be observed. The inset shows the SiO_2_ layer more clearly. (b) TEM image of Ni(Col)/Au@SiO_2_ catalyst/SHINs, showing 4.1 nm Ni NPs adsorbed onto the Au@SiO_2_. (c) TEM image of Ni(Pr)/Au@SiO_2_ catalyst/SHINs, prepared by *in situ* reduction of Au@SiO_2_ SHINs impregnated with a NiCl_2_ precursor to yield small Ni NPs as can be observed on the carbon film. A number of the Ni NPs has been indicated by black arrows. The Ni NP indicated by the asterisk is shown up close in the inset. (d) Scanning Electron Microscopy (SEM) image of the as‐prepared Ni(SA)/Au@SiO_2_ on a Si wafer.

Figure 1 and Figure S1 illustrate the characteristics of the Au@SiO_2_ SHINs used for this study. Transmission Electron Microscopy (TEM) shows nanoparticles (NPs) consisting of a 90 nm diameter Au core coated with a ∼2 nm SiO_2_ shell (Figure 1a). A UV‐Vis absorption band at ∼551 nm originating from the LSPR of the Au NPs can be used to calculate both the average size and concentration of uncoated NPs (Figure S2).^33^ In line with the particle size distribution obtained from TEM measurements, the diameter of the Au NPs was calculated to be 83 nm. The SHINERS‐enhancement and quality of these SHINs was tested using Rhodamine 6G and pyridine as probe molecule, as demonstrated in Figure S3. Rhodamine 6G is a dye molecule with a high Raman cross‐section often used to probe the SERS activity of plasmonic NPs and the subsequent loss in activity upon coating them with dielectric layers.^28^, ^34^, ^35^, ^36^, ^37^ Pyridine is used to detect pinholes present within the SiO_2_ layer surrounding the Au NPs.^34^ As can be observed in Figure S3 upon coating of the Au NPs with SiO_2_, no pyridine signal is observed due to this loss of chemical enhancement. On the same sample we can still detect Rhodamine 6G (Figure S3), indicating that SHINERS‐activity of the materials prepared is not lost.

Figures 1b‐d show TEM and Scanning Electron Microscopy (SEM) images of the Ni/Au@SiO_2_ catalyst/SHINs prepared through the different synthesis methods under study; *i. e*., Precursor (Pr), Colloidal Deposition (CoI) and Spark Ablation (SA). In Figure 1b, Ni(Col)/Au@SiO_2_ with the 4.1 nm Ni NPs on the surface of the Au@SiO_2_ SHINs are shown. Attachment of the Ni NPs via the method of Dong *et al*.^38^ was applied, in which the organic ligand (in this case oleylamine) on the Ni NP surface is exchanged for NOBF_4_, allowing for transfer to aqueous media. Mixing of the ligand‐exchanged Ni NPs and the Au@SiO_2_ SHIN dispersion resulted in spontaneous adsorption of the Ni NPs onto the SHIN surface through electrostatic interactions.^38^, ^39^ Figure 1c shows small 2.6 nm Ni NPs lying close to the Au@SiO_2_ SHINs. Note that this TEM sample was prepared after *in situ* reduction of the NiCl_2_/Au@SiO_2_ catalyst/SHINs by scraping, redispersing, and transferring of the sample from a Si wafer to a Cu grid using isopropanol. Therefore, the lack of Ni NPs on the Au@SiO_2_ surface is not significant as the position of these two types of particles with respect to each other is not representative in the TEM sample. The same method of TEM sample preparation was attempted for Ni(NO_3_)_2_/Au@SiO_2_, Ni(acac)_2_/Au@SiO_2_ (Figure S1a and S1b) and Ni(SA)/Au@SiO_2_ but did not result in clear TEM images. Instead, the as‐prepared Ni(SA)/Au@SiO_2_ samples were investigated with SEM, as presented in Figure 1d. The Au@SiO_2_ SHINs can be observed with bright spots on the surface, which constitute the deposited Ni NPs. Note that due to the (limited) resolution of the SEM image, we cannot investigate the size of the Ni NPs, nor whether these bright spots are agglomerated or aggregated NPs. However, based on extensive testing of the spark ablation setup, individual Ni NPs should be around 2 nm, albeit with a broad size distribution.^32^, ^40^

To confirm the presence of metallic Ni on all Au@SiO_2_ SHINs, *in situ* SHINERS experiments were carried out. The Ni(Col) and Ni(Pr)/Au@SiO_2_ samples had to undergo reduction treatment at high temperature first. Temperature Programmed Reduction (TPR) of the colloidal Ni NPs^41^ gave a required reduction temperature in H_2_ of 450–600 °C to fully reduce the passivated Ni NPs to their metallic phase. However, due to the limited stability of Au@SiO_2_ at high temperatures and the practical limitations of the Linkam Cell brought about by the high heat capacity of H_2_, the maximum temperature that can be reliably achieved is 400 °C. For the precursor samples we have chosen to eliminate a calcination step as the reduction of nickel oxide occurs at even higher temperatures than the direct reduction of the herein used Ni precursors.^42^

**Figure 2:**
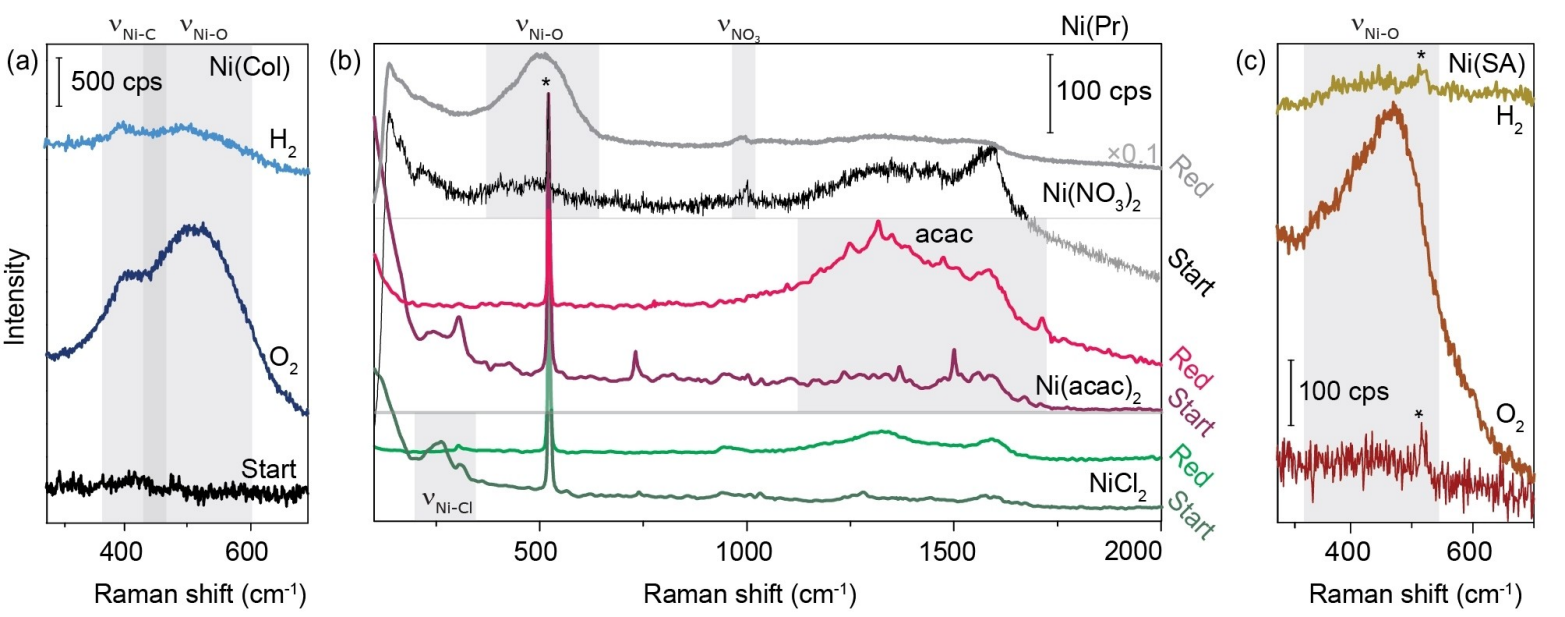
*In situ* Raman spectra during reduction of Ni/Au@SiO_2_ prepared via the Precursor (Pr), Colloidal Deposition (Col) and Spark Ablation (SA) method. (a) Col Ni NPs on Au@SiO_2_ SHINs. First the colloidal Ni NPs were calcined to burn off remnants of the ligands (middle spectrum). This resulted in the appearance of a Ni−O stretching band at ∼515 cm^−1^. The NPs were then subjected to H_2_ at 400 °C in an attempt to obtain metallic Ni (top spectrum). (b) Direct reduction of Ni(Pr)/Au@SiO_2_ at 400 °C Top, black: Ni(NO_3_)_2_/Au@SiO_2_ reduction. Middle, purple: Ni(acac)_2_/Au@SiO_2_ reduction. Bottom: NiCl_2_/Au@SiO_2_. Characteristic bands for the precursor are indicated in the spectra. (c) *In situ* oxidation and reduction of the Ni(SA)/Au@SiO_2_ catalyst/SHINs. Upon oxidation a Raman band at ∼480 cm^−1^ is observed, attributed to the formation of nickel oxide. After reduction with H_2_ at 300 °C for 90 min a weak remnant of the band could still be observed. *Asterisks mark peaks originating from the Si wafer.

*In situ* SHINER spectra of the Ni(Col)/Au@SiO_2_ system in Figure 2a show that we can observe a very weak band for Ni−C stretching at ∼405 cm^−1^ (probably originating from remnants of ligands or solvent molecules on the surface) at the start of the experiment. To remove these ligands from the surface the Ni(Col)/Au@SiO_2_ underwent a calcination/burning step in Ar:O_2_. This treatment resulted in the formation of a new band at ∼515 cm^−1^ assigned to Ni−O stretching, which can subsequently be used to monitor the reduction of the Ni NPs. Upon introduction of reducing environment (Ar : H_2_ 1 : 1 at 400 °C) over time, both the Ni−O and Ni−C stretching vibrations decrease in intensity, indicating the formation of Raman‐inactive, metallic Ni. However, weak Ni−O and Ni−C Raman bands remain visible in the SHINER spectra after H_2_ treatment.

Figure 2b shows the before and after SHINER spectra for the Ni(Pr)/Au@SiO_2_ catalyst/SHIN systems using Ni(NO_3_)_2_ (top), Ni(acac)_2_ (middle) and NiCl_2_ (bottom). The fresh catalysts showed the signals of the corresponding anions and ligands, namely NO_3_ stretching at around 1000 cm^−1^ for Ni(NO_3_)_2_/Au@SiO_2_ and Ni−Cl stretching at approximately 360 cm^−1^ for NiCl_2_/Au@SiO_2_.^17^, ^28^ For Ni(acac)_2_/Au@SiO_2_ some Raman peaks can be observed in the 1250–1600 cm^−1^ region that may originate from the organic anion, but they do not match assignments previously made in literature. Note that the sharp peak at 520 cm^−1^ in the spectra originates from the supporting Si wafer.

Then, the precursors were reduced at 400 °C under H_2_, without a calcination step under O_2_ to prevent the formation of nickel oxide. Still, for Ni(NO_3_)_2_/Au@SiO_2_ the formation of a band attributed to Ni−O stretching vibrations was observed at ∼501 cm^−1^, due to decomposition of the nitrate ions and subsequent formation of nickel oxide.^43^, ^44^ This disqualifies Ni(NO_3_)_2_ as a suitable metal precursor for the preparation of Ni/Au@SiO_2_ catalyst/SHINs, and most probably also for nitrates of other first main transition metal series elements, such as Co.^45^ Reduction of Ni(acac)_2_ on the other hand, shows no Raman peaks that can be attributed to Ni−O^46^ or Ni−C^47^ stretching vibrations, despite the presence of both carbon and oxygen species in acetylacetonate. We do observe decomposition of this coordination complex, responsible for the formation of the so‐called D and G Raman bands at 1320 and 1530 cm^−1^, assigned to disordered and ordered (graphitic) carbonaceous species.^48^ Finally, for NiCl_2_/Au@SiO_2_ we can see a decrease in the intensity of the Raman peak originating from Ni−Cl stretching upon reduction with H_2_.

In contrast, on the Ni(SA)/Au@SiO_2_ sample in Figure 2c initially no Raman bands due to Ni−O or Ni−C were observed. To prove the presence of Ni on the Au@SiO_2_ SHIN surface which could not be unambiguously determined using electron microscopy, the sample was treated with oxygen. Upon introduction of O_2_ into the Linkam Cell, the formation of Raman bands assigned to Ni−O stretching vibrations at ∼480 cm^−1^ could be observed.^46^ Additionally, a shoulder similar in position to that for Ni−C stretching as observed in the Ni(Col)/Au@SiO_2_ sample appeared to form.^47^ This could be due to the Ni interacting with some carbon either still on the SHINs despite the cleaning procedure, or from organics that have adsorbed onto the sample in the glovebox. Reducing these Ni−O and Ni−C species at 300 °C under H_2_ proved challenging: after 2 h Ni−O stretching could still be observed.

Comparing the typical Raman bands of nickel oxide observed in the different SHINER spectra shows that these bands are centered around different Raman shifts, as illustrated in Figure S4. Note that the species found in the colloidal sample upon oxidation and reduction also differ slightly, indicating the existence of more and less stable nickel oxides. Furthermore, the width of the Raman bands is different for the different samples prepared, which has been observed on other transition metals to be a measure of crystallite size and/or lattice strain.^49^, ^50^ A broadening and blue shift of bands was then reported to be an indication of smaller NPs.^28^, ^51^, ^52^ Taking these reports into account, we can conclude that the different preparation methods resulted in the presence of different types of nickel oxides in our samples.

### 2.2 *In Situ* SHINERS Probe Molecules Adsorption and Acetylene Hydrogenation on Ni/Au@SiO_2_ Catalyst/Shell‐Isolated Nanoparticles

**Figure 3:**
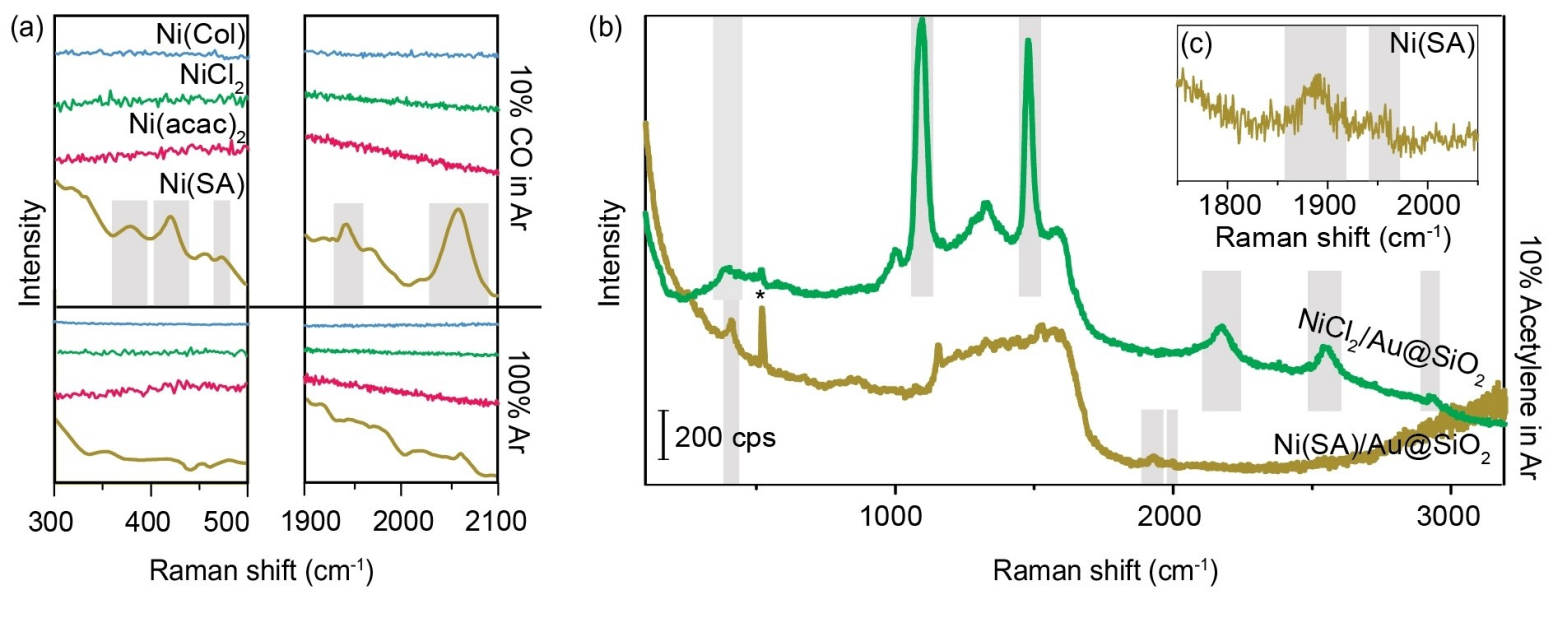
Probe molecule adsorption experiments on Ni/Au@SiO_2_ samples, prepared via the Precursor (Pr), Colloidal Deposition (Col) and Spark Ablation (SA) method. (a) CO adsorption conducted at 150 °C. Bottom spectra in each panel: Ar atmosphere. Top spectra in each panel: 10 % CO in Ar. Blue: Ni(Col)/Au@SiO_2_. Green: NiCl_2_/ Au@SiO_2_. Pink: Ni(acac)_2_/Au@SiO_2_. Brown: Ni(SA)/ Au@SiO_2_. Only for the bottom, brown spectra of Ni(SA)/Au@SiO_2_ some peaks associated with CO adsorption on Ni were observed: at 377, 425 and around 475 cm^−1^ in the Ni−C stretching region, and at 1944 and 2060 cm^−1^ in the C−O stretching region. Based on IR experiments from literature, the peaks in the C−O stretching region are assigned to C−O adsorbed in a bridged and linear configuration, respectively. Note also the difference in the shape of the spectra or baselines before CO adsorption under Ar atmosphere (bottom spectra), which are straight lines for the samples reduced at high T. For the Ni(SA)/Au@SiO_2_ sample this is not the case and some minor bands can be observed. (b) Adsorption of acetylene on (top, green) NiCl_2_/Au@SiO_2_ and (bottom, brown) Ni(SA)/Au@SiO_2_. On both samples, new peaks appear upon the introduction of acetylene, at different Raman shifts. For NiCl_2_/Au@SiO_2_ we observe two very intense peaks at 1095 and 1477 cm^−1^ and their corresponding overtones and combination bands at 2168, 2545 and 2913 cm^−1^. We also see a peak around 400 cm^−1^ in the metal‐adsorbate stretching region. For Ni(SA)/Au@SiO_2_ we observe peaks at different Raman shifts, namely at 411 cm^−1^ in the metal‐adsorbate stretching region, and around 1900 cm^−1^_,_ corresponding to the C≡C stretching region. This region is enlarged in the inset in (c). (c) C≡C stretching region for Ni(SA)/Au@SiO_2_. Two small peaks can be observed at 1890 and 1950 cm^−1^. Note that peaks originating from the Si wafer at 520 cm^−1^ are marked with an *asterisk.

After reduction of the Ni(Pr), Ni(Col) and fresh Ni(SA) materials, CO was introduced into the Linkam Cell to probe the available catalytic surface, similarly to methods we have used before.^30^ Figure 3a shows spectra in the characteristic regions of CO adsorption on metal surfaces for both the low and high wavenumber region, that exhibit metal‐adsorbate and C−O stretching vibrations, respectively. The bottom spectra in the panels of Figure 3a are recorded at 150 °C in 100 % Ar atmosphere before introducing CO, while the top spectra are recorded with 10 % CO in Ar at 150 °C. Figure 3a–c clearly shows that no Raman bands due to CO adsorption on Ni were observed on the Ni(Pr) and Ni(Col) samples. The Ni(SA) sample represented by the bottom spectra, on the other hand, did show characteristics Raman bands for CO adsorption at 150 °C. Two peaks, one at ∼2060 cm^−1^ and one at ∼1944 cm^−1^ attributed to linear and bridged adsorption of CO, respectively can be discerned, which is in line with results from literature on CO adsorption on Ni/SiO_2_.^53^, ^54^, ^55^ In the low wavenumber region, we observe a number of Raman bands that can be attributed to Ni‐CO stretching, like a band at ∼377 cm^−1^, ∼425 cm^−1^ (previously unreported) and ∼475 cm^−1^ (reported both as bending and stretching vibration).^47^, ^56^

Based on the CO adsorption experiments, we postulate that catalytically active Ni NPs were present only in the Ni(SA)/Au@SiO_2_ sample, and not in the Ni(Pr)/Au@SiO_2_ and Ni(Col)/Au@SiO_2_ samples. However, the straight baselines observed for the Ni(Pr)/Au@SiO_2_ and Ni(Col)/Au@SiO_2_ samples as opposed to that of Ni(SA)/Au@SiO_2_ may indicate that upon reduction at high temperature the Au@SiO_2_ SHINs have lost their SERS enhancement. To determine whether we do not observe bands due to a lack of Ni reduction or due to loss of SERS enhancement, the Ni(Pr)/Au@SiO_2_ and Ni(Col)/Au@SiO_2_ samples were also probed with acetylene species. Triple bonds both have a high Raman cross‐section and affinity to metal surfaces and are therefore excellent candidates for *in situ* Raman spectroscopy experiments.

In a separate set of experiments, the activated NiCl_2_/Au@SiO_2_ and as‐prepared Ni(SA)/Au@SiO_2_ were probed with 10 % acetylene in Ar at room temperature, as displayed in Figure 3b. For both samples, this resulted in new Raman peaks, as indicated by the grey bars. Interestingly, the peaks appear at different Raman shifts, namely at ∼1095 and ∼1477 cm^−1^ and corresponding overtones and combination bands at ∼2168, ∼2545, and ∼2913 cm^−1^ for NiCl_2_/Au@SiO_2_. Comparison with literature shows that the Raman peaks reported for the NiCl_2_/Au@SiO_2_ sample originate from acetylene adsorption onto a Au surface or a Au surface with another transition metal in close proximity, and not from adsorption on a (pure) Ni surface.^57^ This is in line with the lack of Ni‐CO stretching vibrations observed and with the aforementioned risk of damaging the isolating oxide coating of the Au@SiO_2_ SHINs during reduction of nickel precursors. To confirm this hypothesis, a pinhole test was conducted after the reduction of NiCl_2_/Au@SiO_2_. The result shown in Figure S7 indeed reveals characteristic peaks for pyridine on a Au surface. A blank acetylene adsorption experiment on Au@SiO_2_ parent SHINs was performed to see if the Au surface was already exposed before the deposition of Ni precursors on the surface, but as displayed in Figure S7, no characteristic acetylene‐Au bands and merely the formation of coke (broad bands at ∼1320 and ∼1530 cm^−1^) was observed. These experiments show that the SERS enhancement of the Au@SiO_2_ SHINs was not lost, but rather that we did not successfully reduce Ni(Pr) in both the CO and acetylene adsorption experiments.

For the Ni(SA)/Au@SiO_2_ sample, the Raman band at ∼1950 cm^−1^ can be attributed to physisorbed acetylene.^58^, ^59^ Next to that, as shown in Figure 3c, a Raman peak was observed at ∼1890 cm^−1^, which has to the best of our knowledge not been reported in literature. Alongside this peak arises a relatively sharp Raman peak in the metal‐adsorbate stretching region, at ∼411 cm^−1^, that we attribute to Ni−C(≡C) stretching in relation to (Ni−)C≡C stretching at ∼1890 cm^−1^. Furthermore, we observe a sharp Raman peak at ∼1136 cm^−1^.

**Figure 4:**
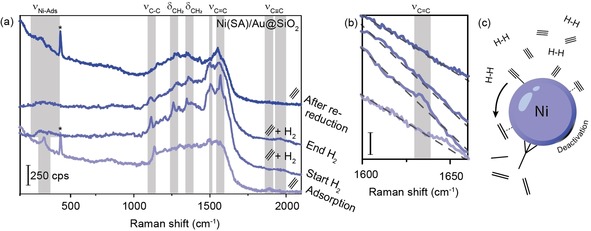
(a) Acetylene and its hydrogenation as observed on Ni(SA)/Au@SiO_2_ at room temperature with SA, Spark Ablation. From bottom to top: upon initial acetylene adsorption, followed immediately by the introduction of hydrogen (“Start H_2_”), after 30 min of hydrogenation (“End H_2_”), and finally after attempted (re‐)reduction of the broad Ni‐adsorbate stretching vibration at 300 °C. Several Raman peaks corresponding to acetylene and related hydrogenated species can be observed. (b) Zoom‐in of the 1600–1660 cm^−1^ region, where free C=C stretching vibrations occur. Upon hydrogenation (second spectrum from the bottom) a shoulder at 1632 cm^−1^ appears, and disappears again with time‐on‐stream (TOS), hinting upon the formation of gas‐phase ethylene. Dashed lines are plotted along the slope to guide the eye. *Asterisks mark peaks originating from the Si wafer. (c) Schematic representation of the species observed with SHINERS during the Ni‐catalyzed hydrogenation of acetylene.

Figure 4a shows the hydrogenation of acetylene at room temperature over the Ni(SA)/Au@SiO_2_ catalyst, with the bottom spectrum showing the adsorption of acetylene as also displayed in Figure 3b. Immediately after adsorption, H_2_ was added to the gas feed (10 % H_2_, 10 % acetylene, 80 % Ar), which resulted in the disappearance of the acetylene‐related Raman peaks at ∼1890 cm^−1^ and ∼411 cm^−1^, and in the appearance of new broad Raman peaks in the metal‐adsorbate stretching region at ∼390 cm^−1^ and ∼450 cm^−1^, and sharp peaks at ∼1261 cm^−1^, ∼1348 cm^−1^, ∼1507 cm^−1^ and ∼1570 cm^−1^. The peak at ∼1136 cm^−1^ either disappears or shifts to ∼1114 cm^−1^; without definite assignment, it is not clear if we are looking at a shift (perhaps due to a coverage effect) or its disappearance and appearance of a new species. Peaks in this range have been observed for the decomposition of ethylene over Pt studied with Electron Energy Loss Spectroscopy (EELS),^60^ for acetylene and ethylene adsorption on Ni/SiO_2_ and Ni/Al_2_O_3_ surfaces with Fourier Transform Infrared (FT‐IR) spectroscopy, and for acetylene adsorbed on Rh (directly deposited on Au electrodes).^57^ In the first, these peaks remain unidentified, in the second they were tentatively assigned to ethylidine species, while in the latter study they are assigned to vibrations resulting from the mixing of C−C single bond stretching and C−H bending. However, these are always paired with only one sharp, intense peak at ∼1500 cm^−1^, which was not unambiguously observed here. Rather, the peak at ∼1507 cm^−1^ that appears upon the introduction of H_2_ seems to have more similarities to π‐bound ethylene on group (I)X transition metals, for which vibrational peaks at ∼1261 cm^−1^ and ∼390 cm^−1^ have been observed as well.^17^, ^61^, ^62^ This indicates that upon hydrogenation of acetylene ethylene is formed. Upon closer inspection of the 1600–1660 cm^−1^ spectral region in Figure 4b, we can observe a small shoulder at ∼1632 cm^−1^ in the first hydrogenation spectrum (2^nd^ from the bottom) that supports this claim, as this Raman band is characteristic for gas‐phase ethylene.^61^

The Raman band at ∼1348 cm^−1^ can be assigned to either symmetric CH_2_ deformation in ethylene or symmetric deformation of CH_3_ for adsorbed ethylidine species, as they occur at similar frequencies. However, based on the simultaneous appearance and disappearance of the ∼1632 cm^−1^ and ∼1348 cm^−1^, we assign the latter to symmetric CH_2_ deformation vibrations.^59^, ^61^, ^62^ Additionally, the band at ∼450 cm^−1^ was also reported for ethylidine on metal surfaces,^61^, ^63^ and this Raman band remains present throughout the hydrogenation experiment (Figure 4a, third spectrum from the bottom, marked “End H_2_”), alongside a band at 1358 cm^−1^ that we therefore propose both originate from ethylidine on a Ni surface. The previously unassigned band in the 1110–1140 cm^−1^ spectral region we can now unambiguously assign to the C−C stretching vibration of ethylidine species on Ni.^59^

Comparing the Raman spectra at the start of the hydrogenation experiment and at the end, after 30 min, we see that the sharp spectral features have broadened significantly. Turning off the acetylene flow and trying to further hydrogenate the adsorbates or (re)reduce the Ni catalyst at 300 °C (which is the upper temperature limit of the Linkam Cell employed) proved unsuccessful, as afterwards broad Ni‐adsorbate species are still observed in the low wavenumber region, whereas the 1100–1600 cm^−1^ spectral region now exhibits more coke‐like features. Upon reintroduction of acetylene into the Linkam Cell, no Raman peaks matching the initial adsorption were observed, including physisorbed acetylene. This indicates deactivation of the Ni catalysts as schematically depicted in Figure 4c, and illustrates again the challenges in working with low transition metal catalysts that require high reduction temperatures. However, the fact that we were successful in studying the *in situ* hydrogenation of acetylene to ethylene over Ni, revealing previously unobserved species, showcases the potential of combining the synthesis method of spark ablation with the SHINERS approach for observing surface reaction intermediates during catalysis.

## 3 Conclusions

We have investigated various synthesis methods [*i. e*. Precursor (Pr), Colloidal Deposition (Col) and Spark Ablation (SA)] to prepare catalytically active nickel catalysts on Au@SiO_2_ for *in situ* SHINERS studies. We demonstrate that the two common synthesis methods (*i. e*. Pr and Col) suitable for making noble metal‐based catalyst materials are not compatible with the SHINERS methodology because of the limitations brought about by the need for a reduction treatment at high temperature. Instead, in this work we propose SA as a useful and novel synthesis method to directly deposit the active metal catalyst on top of Au@SiO_2_ Shell‐Isolated Nanoparticles. We demonstrate that these Ni metal nanoparticles can interact with different adsorbates, such as CO and acetylene. In the adsorption experiments with acetylene, Ni‐acetylene species were observed that have not been reported before. These species disappeared upon hydrogenation, and the Raman bands associated with ethylene and ethylidine on Ni were identified, confirming that *in situ* hydrogenation reactions can be studied over Ni‐based catalysts. As from an economical point of view, metals, like Ni, Co, Cu and Fe, are the preferred ones for making solid catalysts in the chemical industry and therefore this study opens the door to investigating industrially relevant heterogeneous catalysts using the *in situ* SHINERS method.

## Experimental Section

17 nm Au NP seeds were prepared through the Turkevich method^64^ (sodium citrate trihydrate salt, 99 %, Sigma‐Aldrich) reduction of HAuCl_4_ (trihydrate salt, 99.99 %, Alfa Aesar) in MilliPore water (MQ, resistivity 18.2 Ωcm) and subsequently grown to 80–100 nm using NH_2_OH × HCl (99.995 %, Sigma Aldrich) as a mild reducing agent.^65^ Au@SiO_2_ SHINs were prepared following a modified method by the group of Tian,^34^ where silica shell growth took place for 2 days at room temperature^66^ instead of 30 min at 90 °C. The SHINs were characterized by Transmission Electron Microscopy (TEM) on a Tecnai20FEG operated at 200 keV after drying 20 μL on a 300 mesh Cu/Formvar grid (van Loenen Instruments), by UV‐Vis spectroscopy of 2 mL of Au@SiO_2_ SHIN dispersion in MQ in a 10.00 mm quartz cuvette on a Cary 50 UV‐Vis spectrophotometer, and by Raman spectroscopy on a Renishaw InVia confocal microscope equipped with a 200 mW 785 nm laser operated at 1–10 % laser power output, 1200 l/mm grating, and max 1×10 s exposure time. Ni(Pr)/Au@SiO_2_ samples were prepared by dissolving a catalyst precursor (Ni(acac)_2_ (95 %, Sigma‐Aldrich), NiCl_2_ (×6 H_2_O; 99.9 %, Sigma‐Aldrich), Ni(NO_3_)_2_ (×6 H_2_O, 99.99 %, Sigma‐Aldrich) in water and mixing appropriate amounts with Au@SiO_2_ SHINs (concentration from UV‐Vis spectroscopy^33^) to obtain 5–10 wt% Ni/Au@SiO_2_. Ni(Col)/Au@SiO_2_ was prepared by depositing colloidal Ni NPs on Au@SiO_2_.

Following a literature procedure, colloidal Ni NPs were synthesized by reducing the Ni(acac)_2_ precursor with borane *tert*‐butylamine (97 %, Sigma‐Aldrich) in the presence of oleylamine (technical, >70 %, Sigma‐Aldrich) and oleic acid (90 %, Sigma‐Aldrich) under Ar conditions at 90 °C.^67^ A ligand exchange from oleylamine to NOBF_4_ (95 %, Sigma‐Aldrich) was carried out which allowed transfer of the Ni colloids to DMSO (≥99 %, Sigma‐Aldrich).^38^ The colloidal particles in DMSO were then mixed with the Au@SiO_2_ NPs, left to equilibrate and washed to remove unadsorbed Ni NPs.^39^ Subsequently, TEM samples were made by depositing 20 μL on a 300 mesh Cu/Formvar grid (Van Loenen Instruments). For both the Ni(Pr) and Ni(Col)/Au@SiO_2_ samples, 20 μL of the cat/SHIN mixtures was then deposited on a B‐doped Si wafer (Siegert Wafer) to prepare samples for SHINERS experiments. For the Ni(SA) sample, 20 μL of Au@SiO_2_ SHINs was directly deposited on B‐doped Si wafers. These SHINERS samples were then cleaned using the procedure by Hartman *et al*. to get rid of any adsorbates or contaminants.^68^ The Au@SiO_2_ wafers were then mounted in the VSP−G1 nanoparticle generator (VSPARTICLE) and 2 nm Ni NPs were deposited using a diffusion deposition accessory (A1), based on spark ablation as physical production method.^32^ Ni (99.99 %) electrodes were used in an Ar (99.999 %) atmosphere at room temperature and pressure. The Ni NP size is estimated based on a coagulation model developed by VSPARTICLE that uses the ablated material and the gas flow rate and takes polydisperse coagulation and Van der Waals forces into account. The model predicts the mean particle size with 1 nm accuracy.^40^ The A1 deposition accessory was equipped with valves to allow transfer to a N_2_ glovebox and to prevent oxidation of the nanoparticles due to exposure to oxygen from the air. Scanning Electron Microscopy (SEM) on a FEI Helios Nanolab G3 operated at 5–30 keV was used to characterize Ni(SA)/Au@SiO_2_ samples on the B‐doped Si wafers.

For *in situ* SHINERS experiments, Ni(Pr) and Ni(Col)/Au@SiO_2_ samples were loaded in a THMS600 Linkam Cell with a theoretical maximum temperature of 600 °C to allow *in situ* reduction in H_2_:Ar atmosphere. The Ni(SA)/Au@SiO_2_ samples were placed in a glovebox‐compatible THMS300 V Linkam Cell with a maximum set temperature of 300 °C. CO adsorption SHINERS experiments for all samples and acetylene adsorption on Ni(Pr) and Ni(SA) samples were carried out using a Horiba XPlora microscope equipped with 638 and 785 nm lasers. Phenylacetylene (98 %, Sigma‐Aldrich) adsorption experiments were carried out on a Renishaw InVia Confocal Raman microscope equipped with a 785 nm laser for Ni(Col)/Au@SiO_2_. Catalytic tests were carried out using the adsorption and hydrogenation of acetylene over Ni(SA)/Au@SiO_2_ samples. After SHINERS experiments, TEM samples of Ni(Pr)/Au@SiO_2_ were prepared by redispersion of the deposited samples in EtOH (99.5 %, ACROS Organics)/iPrOH (≥98 %, Sigma‐Aldrich) and transferring them to 300 mesh Cu/Formvar grids (Van Loenen Instruments)..

## Supporting information

As a service to our authors and readers, this journal provides supporting information supplied by the authors. Such materials are peer reviewed and may be re‐organized for online delivery, but are not copy‐edited or typeset. Technical support issues arising from supporting information (other than missing files) should be addressed to the authors.

Supplementary
