# Supplementary material for: In Situ Shell‐Isolated Nanoparticle‐Enhanced Raman Spectroscopy of Nickel‐Catalyzed Hydrogenation Reactions
Source: Chemphyschem. 2020 Feb 4;21(7):625–32. doi: 10.1002/cphc.201901162 (PMC7187311; doi:10.1002/cphc.201901162)
Supplement: Supplementary file 1 — Supplementary [file CPHC-21-625-s001.pdf]

CHEMPHYSCHEM

Supporting Information

In Situ Shell-Isolated Nanoparticle-Enhanced Raman Spectroscopy of Nickel-Catalyzed Hydrogenation Reactions

Caterina S. Wondergem, Josepha J. G. Kromwijk, Mark Slagter, Wilbert L. Vrijburg, Emiel J. M. Hensen, Matteo Monai, Charlotte Vogt, and Bert M. Weckhuysen*© 2020 The Authors. Published by Wiley-VCH Verlag GmbH & Co. KGaA.
This is an open access article under the terms of the Creative Commons Attribution License, which permits use, distribution and reproduction in any medium, provided the original work is properly cited.

Supporting Information

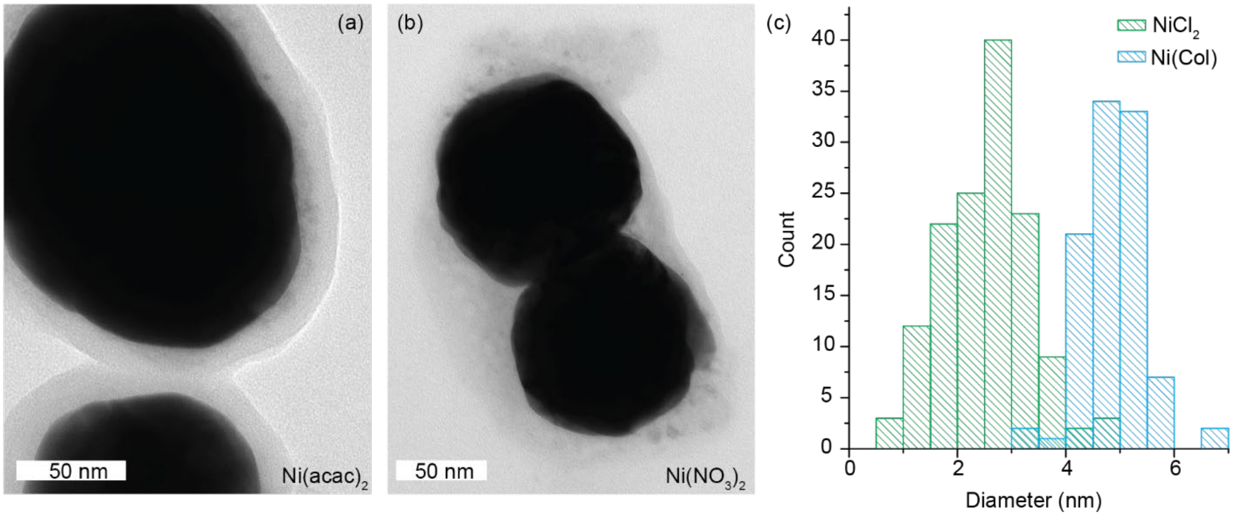

Figure S1 Transmission Electron Microscopy (TEM) characterization of Ni/Au@SiO_2 catalyst/Shell-Isolated Nanoparticles (SHINs) used in this study. (a) Ni/Au@SiO_2 prepared from Ni(acac)$_2$. After *in situ* reduction the SiO_2 layer around the Au NPs seems to have increased in thickness. This apparent increase in thickness is attributed to the decomposition of the organic acac anion, resulting in a layer of carbon - with TEM contrast similar to SiO_2 - around the Au@SiO_2 SHINs. Some small Ni NPs can be observed within this layer. (b) Ni/Au@SiO_2 prepared from Ni(NO_3)$_2$. After *in situ* reduction some spots of varying size with higher contrast can be observed surrounding the Au@SiO_2 SHINs, which are assumed to be Ni(O) NPs. (c) Size distributions for Ni NPs prepared from $NiCl_2$ (green) and colloidal Ni NPs (blue). *In situ* reduction of the $NiCl_2$ precursor resulted in an average size of 2.6 ± 0.8 nm based on 138 NP. The Ni colloids had an average size of 4.1 ± 0.5 nm. On the samples in (a) and (b) we did not observe sufficient Ni NPs to obtain a size distribution.

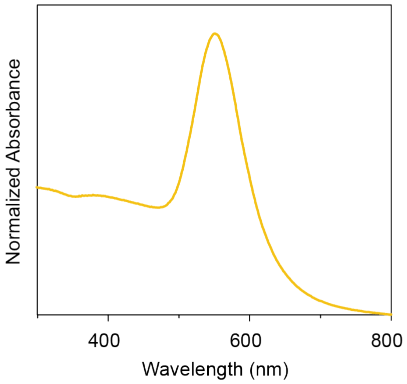

Figure S2 UV-Vis spectroscopy of parent Au NPs. A UV-Vis absorption band at ~ 551 nm originating from the Localized Surface Plasmon Resonance (LSPR) of the Au NPs can be used to calculate both the average size and concentration of uncoated NPs (Figure 1b).[1] In line with the particle size distribution obtained from Transmission Electron Microscopy (TEM) measurements, the diameter of the Au NPs was calculated to be 83 nm.

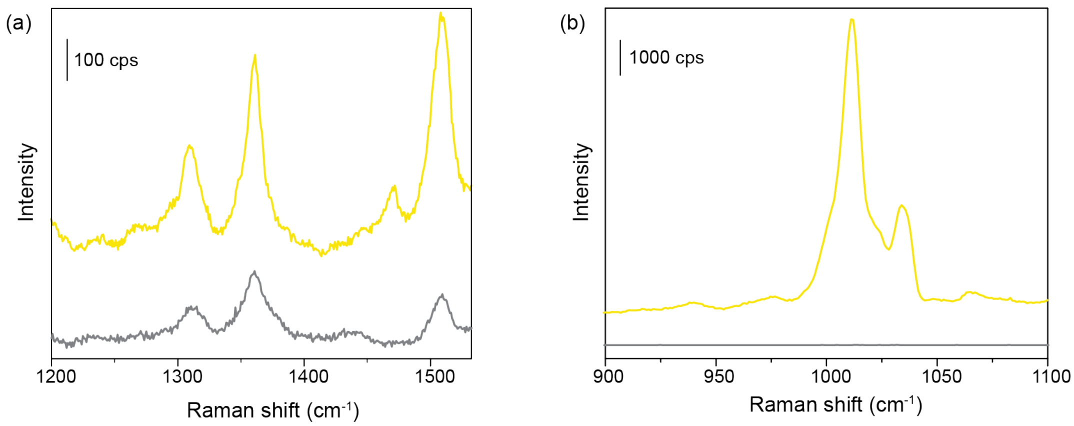

Figure S3 Shell-Isolated Nanoparticle-Enhanced Raman Spectroscopy (SHINERS) activity and pinhole tests of Au@SiO_2 Shell-Isolated Nanoparticles (SHINs). The SHINERS-enhancement and quality of the SHINs was tested using Rhodamine 6G (a) and pyridine (b). Rhodamine 6G is a dye molecule with a high Raman cross-section often used to probe the SERS activity of plasmonic NPs and the subsequent loss in activity upon coating them with dielectric layers.[2–6] The spectra in (a) show that upon coating the Au NPs (yellow, top) with SiO_2 (grey, bottom) the Rhodamine 6G signal goes down. The spectra in (b) show pyridine tests: Pyridine is used to detect pinholes in the SiO_2 layer around the Au NPs.[3] Its lone pair can coordinate to the Au NP surface, which gives rise to chemical enhancement of the signal as well as traditional enhancement. Upon coating of the Au NPs (yellow, top) with SiO_2 (grey, bottom), the pyridine signal cannot be observed due to this loss of chemical enhancement.

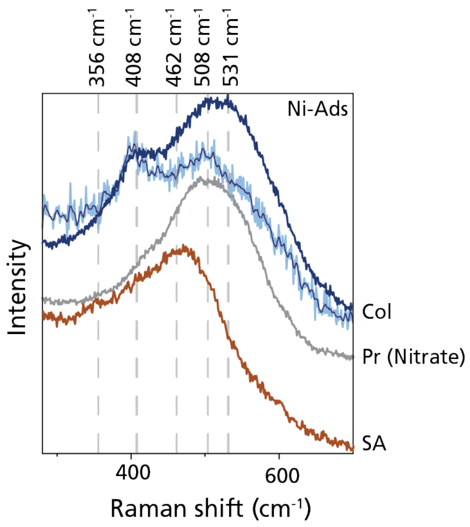

Figure S4 Comparison of the different Ni-adsorbate stretching vibrations observed on the oxidized Ni/Au@SiO_2 samples. Dark blue: Oxidized Ni(Col), light blue: (smoothed to guide the eye, intensified for clarity) reduced Ni(Col), grey: reduced $Ni(NO_3)_2$, brown: oxidized Ni(SA). Interestingly, the Raman band positions for the oxidized species differ, shifting from ~ 530 cm^{-1} on Ni(Col) to ~ 470 cm^{-1} on Ni(SA). Furthermore, for Ni(SA) an unknown band at 360 cm^{-1} can be observed. Note that the species found in the colloidal sample upon oxidation and reduction also differ slightly, indicating the existence of different types of nickel oxides. Furthermore, the width of the Raman bands is different for the different samples, which has been observed on other transition metals to be a measure of crystallite size and/or lattice strain, [7,8] with a broadening and blue shift of bands reported to be an indication of smaller NPs.[2,9,10] Therefore we can conclude that the different synthesis methods yield different types of Ni(O) NPs.

In Situ SHINERS and DRIFTS of Acetylene Species on Ni(Col)/Au@SiO_2

Figure S5 shows the results of the phenylacetylene species adsorption experiments. First of all, it seems like phenylacetylene adsorbs onto the Ni(Col) surface as characteristic peaks for phenylacetylene are observed. Comparison with spectra obtained of phenylacetylene adsorption on Pt as we recently published[11], shows similar characteristic vibrations, like ring vibrations and

C=C stretching vibrations at 1000 cm^{-1} and 1580 cm^{-1} respectively. However, close inspection of the C≡C stretching region only reveals one peak at 2105 cm^{-1}. This peak originates from a C≡C with terminal hydrogen, like observed in the reference spectrum (red spectrum) and not chemisorbed onto a metal surface. The low intensity of the peak compared to the reference spectrum instead indicates physisorption of phenylacetylene on the Ni(Col)/Au@SiO_2 catalyst/SHINs, which may be due to the absence of metallic surface Ni.

To investigate the interaction between phenylacetylene and the nickel colloids in more detail, the colloidal Ni catalysts were deposited on a DAVICAT SiO_2 support following the procedure by Casavola *et al*.[12] and phenylacetylene adsorption on the Ni/SiO_2 catalyst was investigated using Diffuse Reflectance InfraRed Fourier Transform Spectroscopy (DRIFTS). This technique allows us to derive complementary information, especially regarding C-H stretching vibrations, which are insufficiently enhanced in the SHINER spectra to use for characterization. Additionally, this experimental setup does not

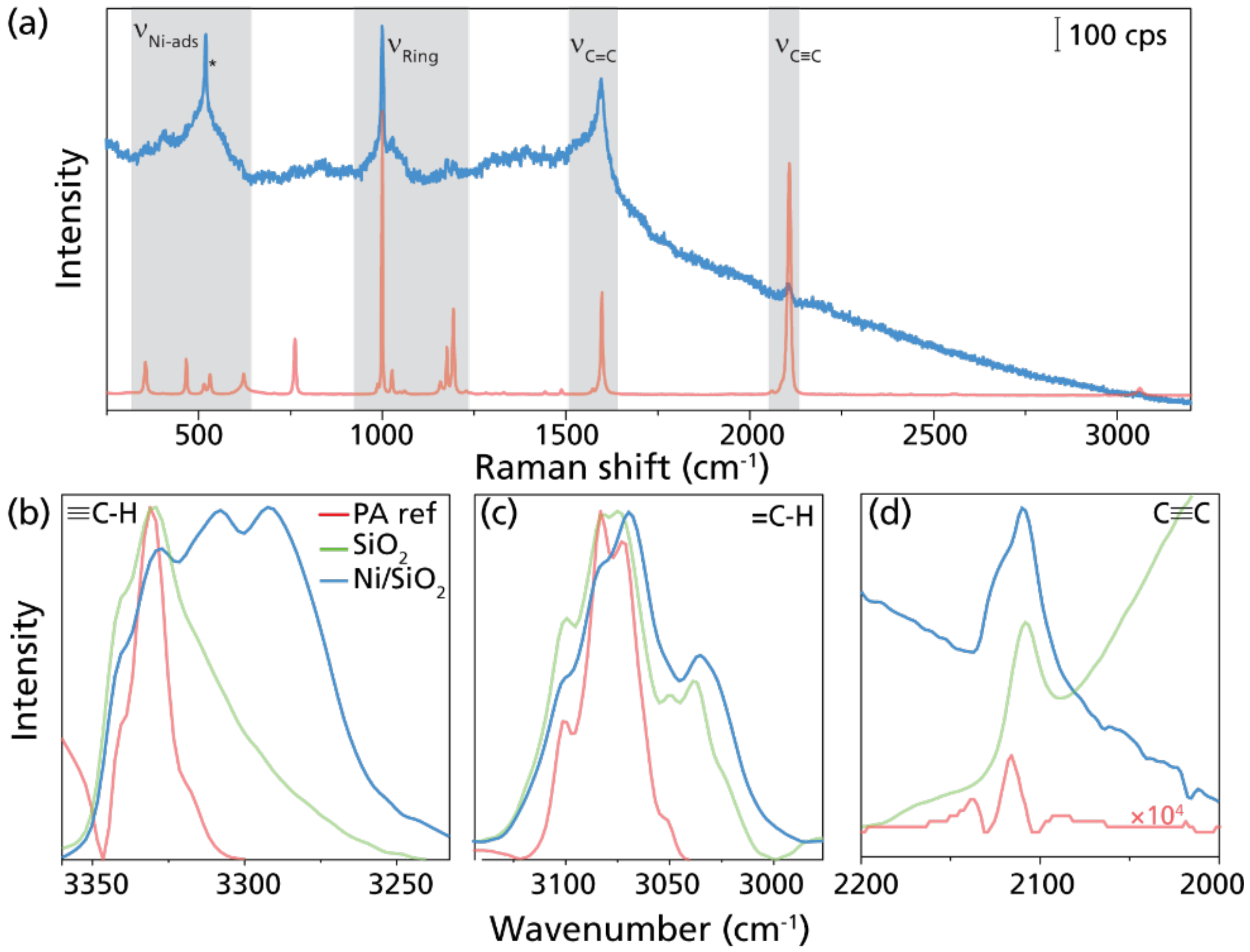

Figure S5. Phenylacetylene probe experiments on colloidal Ni. (a) Shell-Isolated Nanoparticle-Enhanced Raman (SHINER) spectrum of phenylacetylene adsorption on Ni(Col)/Au@SiO_2 after apparent reduction (blue) and reference Raman spectrum of pure phenylacetylene (red). Characteristic peaks for phenylacetylene are observed as indicated in the spectrum. The broad band around 510 cm^{-1} observed underneath the Si wafer (marked with an *asterisk) is indicative of the presence of nickel oxide, as was observed in Figure 5.2. (b-d) Diffuse Reflectance Infrared Fourier-Transform Spectra of gaseous phenylacetylene (red), and adsorbed on SiO_2 (green) and Ni/SiO_2 (blue). (b) C≡C-H stretching region showing a narrow sharp peak for free phenylacetylene. Upon physisorption on SiO_2 the peak broadens a bit. Upon adsorption on the Ni/SiO_2 catalyst we see new peaks arising at lower wavenumbers, indicating a weakening of the bond. (c) C=C-H stretching region, showing the vibrations arising from the phenyl ring. Again the largest difference is observed for the Ni/SiO_2 catalyst, indicating that there is interaction between the ring and the Ni surface. (d) C≡C stretching region. Upon adsorption of phenylacetylene on Ni/SiO_2 a second peak can be observed in the shape of a shoulder. Note that due to the IR inactivity of the C≡C stretching mode the peak in the red reference spectrum is very weak. The intensity of this peak in the other spectra indicate that either more phenylacetylene is present in total, giving a stronger signal, or that upon interaction with SiO_2 and Ni the stretching mode changes to such an extent that it becomes more IR-active.

require Au NPs to enhance the signal and employs a different *in situ* cell, the both of which allow for full reduction of the Ni NPs at the temperature determined by Vrijburg *et al.*[13]

After reduction of the catalysts at 500 °C for 1 h in a 1:1 H_2:He gas feed, the Ni/SiO_2 catalysts were subjected to phenylacetylene vapor saturated in N_2. A blank experiment with just SiO_2 was carried out as well. The DRIFT spectra are displayed in Figure S5b-d, showing the different spectral regions for characteristic phenylacetylene vibrations that can be observed with IR. First of all, in the (C≡)C-H stretching region in Figure S5b, we can see a narrow, sharp peak for the reference DRIFT spectrum of free phenylacetylene (red). On a blank, dried SiO_2 reference sample, we see some broadening of this peak (green), whereas on Ni/SiO_2 (blue) we see distinct new maxima at lower wavenumbers, indicating interaction between the acetylene group and the Ni surface. The (C=)C-H stretching region in Figure S5c shows the C-H vibrations from the aromatic ring. Compared to the red reference spectrum for free, gas phase phenylacetylene, we see new peaks in both the SiO_2 and Ni/SiO_2 samples at lower wavenumbers. This either indicates interaction with the phenyl ring, the occurrence of dissociative adsorption in which the acetylene group becomes more ethylene-like, or a combination of both. Finally, in the C≡C stretching region displayed in Figure S5c, we see the symmetric stretching vibration of the acetylene group at around 2115 cm^{-1}. For the reference spectrum this vibration is very weak (spectrum multiplied by 10^4 × for clarity), as this vibration does not fulfill the selection rules for IR activity (change in dipole moment).[14] However, when phenylacetylene is measured on SiO_2 and Ni/SiO_2 this peak becomes much more intense due to distortion of the bond and related increased IR activity. Furthermore, the peak shifts to lower wavenumbers (2110 cm^{-1}) and an extra peak is observed on the Ni/SiO_2 sample as a shoulder, all indicating direct interaction and chemisorption of phenylacetylene through the acetylene group on activated, metallic Ni catalysts.

Based on these DRIFTS results, we can say that in the SHINER spectrum in Figure S5a, no active Ni surface was present or accessible to phenylacetylene, resulting in only physisorption on either unreduced Ni surface or the SiO_2 shell. Comparison of the metal-adsorbate stretching region in the SHINER spectra in Figures 2 of the main text and Figure S5a show a broad band around 500 cm^{-1}, that points towards the presence of nickel oxide species. This is in line with incomplete reduction of the Ni(Col)/Au@SiO_2 catalyst/SHINs, and further confirms the difficulty of using the colloidal deposition method for the preparation of active Ni/Au@SiO_2 samples for *in situ* SHINERS studies.

Preparation of Ni/SiO_2 samples for DRIFTS experiments

Ni/SiO_2 samples were prepared by the method of Casavola *et al.*[12] In short, ~800 mg DAVICAT® SI 1302 Silica Powder was mixed with a dispersion of 60 mg Ni colloids in 3 mL toluene (99+%, ACROS Organics) and 5 mL 1-octadecene (>90%, Sigma-Aldrich) while stirring. The NP/SiO_2 mixture was degassed and put under vacuum. After evaporation of toluene, the mixture was heated to 120 °C and kept at this T for 30 min. The mixture was flushed with N_2 three times and heated to 300 °C for 60 min. After cooling down to room temperature, the Ni/SiO_2 catalyst was washed alternatingly with *n*-hexane (99+%, ACROS Organics) and acetone (99.6%, ACROS Organics), several times. The samples were dried at 60 °C overnight, then 120 °C for 3 h and finally at 80 °C under vacuum for 3 h. A TEM sample is included in Figure S6.

DRIFTS experiments were carried out on a Bruker Tensor 27 FT-IR spectrometer equipped with an MCT detector, a Praying Mantis diffuse reflectance accessory, and a high temperature reactor cell with a KBr window. The Ni/SiO_2 powder samples were loaded into the sample cup of the reactor cell, packed on a quartz wool bed. The reactor cell was heated by an automatic temperature controller (Harrick ATC-02402). Spectra were recorded at a spectral resolution of 4 cm^{-1} over a range from 4000-600 cm^{-1}.

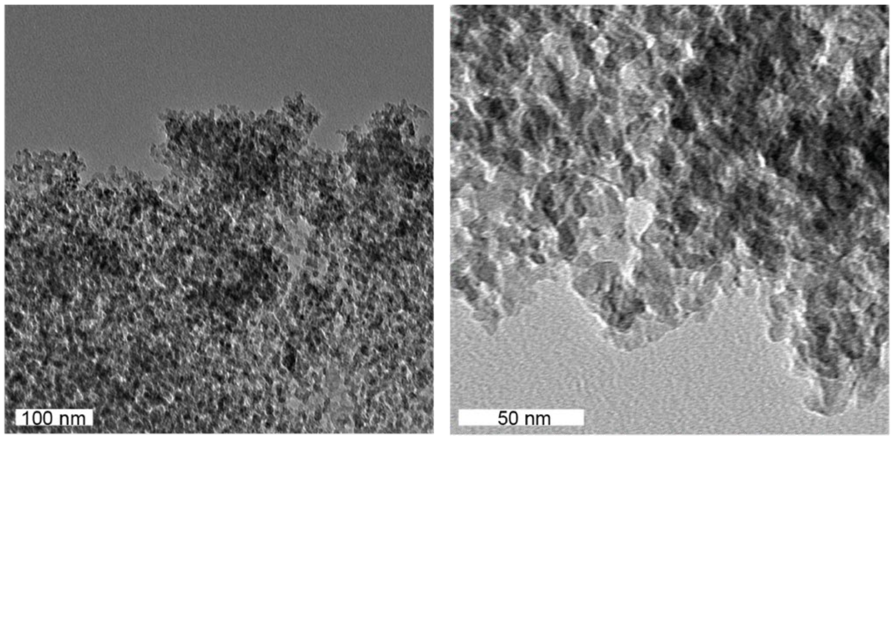

Figure S6 Transmission Electron Microscopy (TEM) images of Ni(Col)/SiO_2. Due to the similar structure of DAVICAT SiO_2 and the colloidal Ni NPs the Ni NPs are hard to observe.

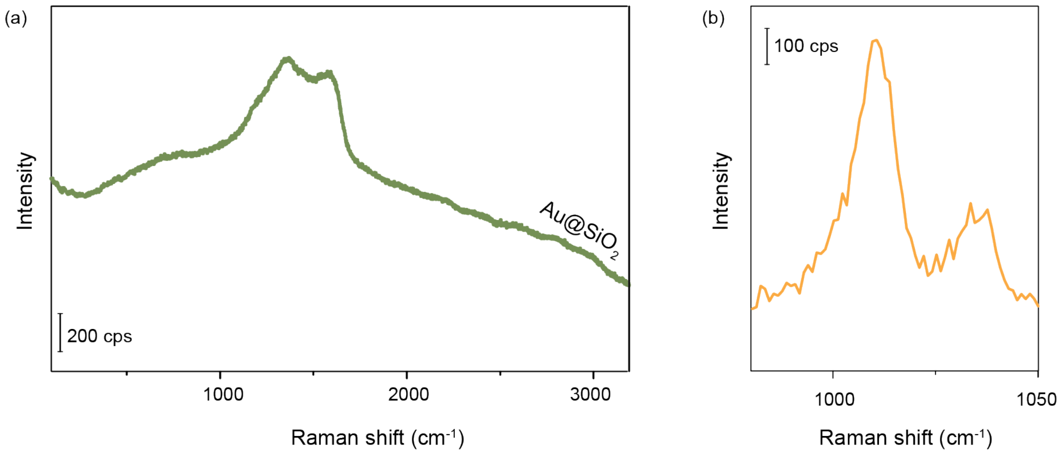

Figure S7. (a) Shell-Isolated Nanoparticle-Enhanced Raman (SHINER) spectra of acetylene adsorption on Au@SiO_2 SHINs after undergoing the same treatment as impregnated Ni/Au@SiO_2 samples. No Raman peaks due to adsorption of acetylene on Au were observed. Only the formation of coke (between 1200-1600 cm^{-1}) was observed. (b) Pyridine/pinhole test on $NiCl_2$/Au@SiO_2 catalyst/SHINs after reduction. Raman peaks pointing to the presence of pyridine adsorbed on a bare Au surface can be observed at 1000 cm^{-1}, indicating the SiO_2 shell now contains pinholes.

References

(1) Haiss, W.; Thanh, N. T. K.; Aveyard, J.; Fernig, D. G. Determination of Size and Concentration of Gold Nanoparticles from UV − Vis Spectra. *Anal. Chem.* **2015**, *79*, 4215–4221.

(2) Hartman, T.; Weckhuysen, B. M. Thermally Stable TiO_2- and SiO_2 -Shell-Isolated Au Nanoparticles for In Situ Plasmon-Enhanced Raman Spectroscopy of Hydrogenation Catalysts. *Chem. Eur. J.* **2018**, *24*, 3734–3741.

(3) Li, J. F.; Tian, X. D.; Li, S. B.; Anema, J. R.; Yang, Z. L.; Ding, Y.; Wu, Y. F.; Zeng, Y. M.; Chen, Q. Z.; Ren, B.; Wang, Z. L.; Tian, Z. Q. Surface Analysis Using Shell-Isolated Nanoparticle-Enhanced Raman Spectroscopy. *Nat. Protoc.* **2013**, *8*, 52–65.

(4) Harvey, C. E.; Van Schrojenstein Lantman, E. M.; Mank, A. J. G.; Weckhuysen, B. M. An Integrated AFM-Raman Instrument for Studying Heterogeneous Catalytic Systems: A First Showcase. *Chem. Commun.* **2012**, *48*, 1742–1744.

(5) Hildebrandt, P.; Stockhurger, M. Surface-Enhanced Resonance Raman Spectroscopy of Rhodamine 6G Adsorbed on Colloidal Silver. *J. Phys. Chem.* **1984**, *88*, 5935–5944.

(6) Beer, D.; Weber, J. Photobleaching of Organic Laser Dyes. *Opt. Commun.* **1972**, *5*, 307–309.

(7) Dohcevic-Mitrovic, Z. D.; Šćepanović, M. J.; Grujić-Brojčin, M. U.; Popović, Z. V.; Bošković, S. B.; Matović, B. M.; Zinkevich, M. V.; Aldinger, F. The Size and Strain Effects on the Raman Spectra of $Ce_{1-X}Nd_XO_{2-\delta}$ ($0 \leq x \leq 0.25$) Nanopowders. *Solid State Commun.* **2006**, *137*, 387–390.

(8) Yang, L.; Cui, X.; Zhang, J.; Wang, K.; Shen, M.; Zeng, S.; Dayeh, S. A.; Feng, L.; Xiang, B. Lattice Strain Effects on the Optical Properties of MoS_2 Nanosheets. *Sci. Rep.* **2014**, *4*, 5649.

(9) Jo, H. C.; Kim, K. M.; Cheong, H.; Lee, S.-H.; Deb, S. K. In Situ Raman Spectroscopy of $RuO_2 \cdot H_2O$. *Electrochem. Solid-State Lett.* **2005**, *8*, E39–E41.

(10) Mar, S. Y.; Chen, C. S.; Huang, Y. S.; Tiong, K. K. Characterization of RuO_2 Thin Films by Raman Spectroscopy. *Appl. Surf. Sci.* **1995**, *90*, 497–504.

(11) Wondergem, C. S.; Hartman, T.; Weckhuysen, B. M. In Situ Shell-Isolated Nanoparticle-Enhanced Raman Spectroscopy to Unravel Sequential Hydrogenation of Phenylacetylene over Platinum Nanoparticles. *ACS Catal.* **2019**, 10794–10802.

(12) Casavola, M.; Hermannsdörfer, J.; De Jonge, N.; Dugulan, A. I.; De Jong, K. P. Fabrication of Fischer-Tropsch Catalysts by Deposition of Iron Nanocrystals on Carbon Nanotubes. *Adv. Funct. Mater.* **2015**, *25*, 5309–5319.

(13) Vrijburg, W. L.; Van Helden, J. W. A.; Van Hoof, A. J. F.; Friedrich, H.; Groeneveld, E.; Pidko, E. A.; Hensen, E. J. M. Tunable Colloidal Ni Nanoparticles Confined and Redistributed in Mesoporous Silica for CO_2 Methanation. *Catal. Sci. Technol.* **2019**, *9*, 2578–2591.

(14) Socrates, G. *Infrared and Raman Characteristic Group Frequencies*, 3rd ed.; John Wiley & Sons Ltd: Chichester, 2001.
